# Taxonomic Profiling of Systemic Inflammatory Parameters as Predictors of Tumor Progression in Primary Colorectal Cancer

**DOI:** 10.3390/jcm14248733

**Published:** 2025-12-10

**Authors:** Michał Nycz, Dariusz Waniczek, Małgorzata Muc-Wierzgoń, Karolina Snopek-Miśta, Mariusz Kryj, Bartosz Bichalski, Magdalena Bichalska-Lach, Łukasz Michalecki, Wiktor Krawczyk, Zbigniew Lorenc

**Affiliations:** 1 Department of Oncological Surgery, Faculty of Medical Sciences in Zabrze, Medical University of Silesia, 40-055 Katowice, Poland; michaltnycz@gmail.com (M.N.); dwaniczek@sum.edu.pl (D.W.); karolinasnopek@gmail.com (K.S.-M.); mariusz.kryj@sum.edu.pl (M.K.); 2 Department of Internal Diseases Propaedeutics and Emergency Medicine, Faculty of Public Health in Bytom, Medical University of Silesia, 40-055 Katowice, Poland; 3 Doctoral School, Medical University of Silesia, 40-055 Katowice, Poland; bichalski@wp.pl; 4 Department of Surgical Nursing and Propaedeutics of Surgery, Faculty of Health Sciences in Katowice, Medical University of Silesia, 40-055 Katowice, Poland; mbichalska@sum.edu.pl; 5 Department of Radiotherapy, Faculty of Medical Sciences in Zabrze, Medical University of Silesia, 40-055 Katowice, Poland; lukasz.michalecki@sum.edu.pl; 6 Clinical Department of General and Colorectal Surgery and Multiple Trauma, Medical University of Silesia, 40-055 Katowice, Poland; w.krawczyk@interia.pl (W.K.); zlorenc@sum.edu.pl (Z.L.)

**Keywords:** colorectal cancer, systemic inflammation, taxonomic classification, neutrophil-to-lymphocyte ratio, platelet-to-lymphocyte ratio, prognosis

## Abstract

**Background/Objectives:** Colorectal cancer (CRC) is one of the most common malignancies worldwide, with systemic inflammation increasingly recognised as a determinant of disease progression. This study aimed to establish a taxonomy-based classification of patients with newly diagnosed primary CRC using systemic inflammatory, haematological, and anthropometric markers, and to evaluate its association with tumour stage. **Methods:** A total of 229 patients (111 women, 118 men) undergoing surgery for primary CRC were included. Blood samples were analysed for haemoglobin, leukocytes, neutrophils, lymphocytes, platelets, C-reactive protein (CRP), and carcinoembryonic antigen (CEA). Anthropometric data were collected. Taxonomic clustering and ordinal logistic regression were used to explore associations with TNM and Astler–Coller classifications. **Results:** Men had higher neutrophil and leukocyte counts, elevated CEA concentrations (132.8 vs. 81.3 ng/mL), and higher NLR values (4.74 vs. 4.23) compared with women. Logistic regression confirmed that platelet count (OR 1.003; *p* = 0.004), PLR (OR 1.003; *p* = 0.003), and CEA (OR 1.03; *p* < 0.001) were positively associated with advanced TNM stage, while haemoglobin was inversely correlated (OR 0.88; *p* = 0.045). Among 84 clustering models, two taxonomies were the most clinically informative: Taxonomy I (BMI, neutrophils, platelets) and Taxonomy II (age, lymphocytes, platelets), both significantly associated with T, N, M, overall TNM stage, and Astler–Coller grade. Taxonomy I identified three patient groups. Type 3 represented the poorest phenotype, characterised by low BMI and haemoglobin, high platelets, elevated CEA and PLR, and predominance of TNM IIIC tumours, consistent with a cachectic–inflammatory profile. Type 1 displayed higher BMI, lower inflammation, and earlier-stage disease. Type 2 was characterized by elevated neutrophils and leukocytes. Taxonomy II distinguished four groups, with Type 2 demonstrating the most favourable profile (high haemoglobin and lymphocytes, low NLR and PLR, early TNM stage). **Conclusions:** Systemic inflammatory markers, haemoglobin, platelets, and CEA strongly predict CRC advancement. The proposed taxonomy provides clinically meaningful stratification of CRC patients and may support personalised risk assessment. This accessible approach may facilitate early identification of high-risk individuals, although validation in prospective studies is required.

## 1. Introduction

The diagnosis and treatment of cancer remain among the key challenges of contemporary medicine, with colorectal cancer (CRC) holding a particularly important position. CRC is characterized by high incidence and mortality rates and is a major focus of oncological research worldwide. The global number of new CRC cases is projected to reach 3.2 million by 2040, driven by population aging, growth, and changes in human development [1]. Furthermore, increases in CRC mortality among young adults are projected in several countries, including Italy, the United Kingdom, Poland, and Spain for men, and Germany for women [2].

Given the biological heterogeneity of CRC and the complexity of its progression, identifying novel diagnostic and prognostic tools remains an urgent priority. Increasing attention is being paid to inflammatory markers and indicators of systemic immune response as promising prognostic and predictive factors [3].

Chronic inflammation plays a significant role in the pathogenesis of many tumors, including CRC. It contributes to cancer cell proliferation, angiogenesis, and metastatic potential. Analysis of inflammatory processes and their associated molecular signatures offers valuable insight into mechanisms of carcinogenesis, tumor invasiveness, and response to treatment [4,5,6].

Among the most accessible and clinically relevant prognostic markers are hematological indicators derived from peripheral blood. The neutrophil-to-lymphocyte ratio (NLR) is considered a particularly strong predictor of overall survival (OS) and disease-free survival (DFS) in CRC patients [7,8]. A meta-analysis showed that NLR values ≥ 3.0 and platelet-to-lymphocyte ratio (PLR) ≥ 160 were associated with poor prognosis in stage III–IV CRC [9]. Moreover, recent research has demonstrated that pre-diagnostic levels of neutrophils, leukocytes, and NLR also serve as independent predictors of CRC outcomes, even before clinical diagnosis [10].

Other markers such as PLR, lymphocyte-to-monocyte ratio (LMR), and the systemic immune–inflammation index (SII) have also shown prognostic value in various stages of CRC, particularly in the postoperative setting [11,12]. Inflammation-related plasma proteins such as C-reactive protein (CRP), IL-6 (interleukin-6), TNF-alpha (tumor necrosis factor) and its receptors, adiponectin play complex roles in colorectal cancer development [13].

Understanding the role of inflammation in CRC development and progression is essential for refining risk stratification, improving early detection, and identifying potential targets for immunomodulatory therapies. Consequently, the investigation of inflammatory markers remains a dynamic and clinically relevant field in modern oncology.

Actually, CRC is the second most common cancer in women and the third in men in Poland [14,15,16]. In 2022, the number of new CRC cases exceeded 18,800, with an incidence rate of 57 per 100,000 in men and 44 per 100,000 in women [17]. Despite detailed consideration of the epidemiology of this pathology, prognostic interventions in this area were not discussed.

Some authors have considered CRC to be a “disease of affluence,” as its prevalence and dynamics are closely related to the level of development and living conditions in different regions worldwide [18,19]. Currently, the highest increase in cancer incidence is observed in countries where economic development has been the most rapid in recent decades.

Although the socioeconomic background of CRC is increasingly recognised, current prognostic approaches still rely largely on clinical staging and histopathological assessment. Meanwhile, growing evidence suggests that systemic inflammation plays a central role not only in tumorigenesis but also in tumour progression and patient prognosis. Therefore, accessible biomarkers reflecting the systemic immune response—such as leukocyte, neutrophil, lymphocyte, and platelet counts—are being actively explored as potential tools to support clinical decision-making [20]. Despite their simplicity, these markers may offer valuable insights into disease aggressiveness and progression.

The role of body mass index (BMI) in CRC progression is still under debate. While obesity is a recognized risk factor for CRC development, its relationship with tumour stage at diagnosis remains inconclusive. Some studies suggest that higher BMI may not correlate with more advanced TNM (tumor, nodes, metastases) stages, possibly due to earlier detection in overweight individuals or the so-called “obesity paradox” [20,21]. Conversely, underweight status and sarcopenia have been associated with more aggressive disease and poorer outcomes [22,23]. Furthermore, BMI does not reflect body fat distribution, which may be more relevant for cancer biology. Therefore, assessing the prognostic value of BMI alongside inflammatory markers may contribute to a more comprehensive understanding of disease progression in CRC.

By focusing on routinely measured hematological parameters and their correlation with tumour stage, the aim of the present study was to develop a clinical-biochemical taxonomy of patients with newly diagnosed primary colorectal cancer (CRC), identifying distinct taxa based on systemic inflammatory, hematological, and anthropometric markers, and to assess their relationship with tumour stage and disease progression.

## 2. Materials and Methods

### 2.1. Materials

A total of 229 patients with primary CRC who underwent surgical treatment at two departments of General and Colorectal Surgery in Poland (Sosnowiec, Bytom) were included. A comprehensive medical interview and physical examination were performed for each participant. Clinical data were obtained using a physician-administered questionnaire and included: clinical symptoms and their duration, body mass index (BMI; including weight loss within the previous three months), general condition assessed according to the Karnofsky performance status [23], family history of cancer, comorbidities, and tumor location. Furthermore, the exclusion criteria were as follows: emergency hospitalization, lack of histological confirmation of cancer, a concomitant inflammatory disease, previous non-adjuvant therapy, and no written informed consent.

The study was conducted in accordance with the Declaration of Helsinki and approved by the Bioethics Committee of the Medical University of Silesia. As this was a retrospective analysis based on anonymized clinical data, formal bioethical committee approval was not required. (application no. KNW/0022/KB/74/I/19, 21 March 2019). Statistical analyses were independently verified by consulting biostatisticians from the Department of Public Health, Wroclaw Medical University.

### 2.2. Methods

#### 2.2.1. Patient’s Database Completing

The database also contained information on surgical interventions and histological findings. The evaluation criteria included parameters such as the TNM classification [24], the number of lymph nodes removed and their involvement in the cancer process, the degree of histological differentiation, stage, and the degree of clinical progression according to the TNM and Astler-Coller classification [25].

#### 2.2.2. Analysis of Blood Samples

All venous blood samples were collected in the morning after an overnight fast and processed in a certified hospital laboratory according to standard clinical procedures.

Although routine biochemical and hematological panels were performed for all patients, the present analysis focused on selected parameters relevant to systemic inflammation and tumour progression. These included: hemoglobin, leukocytes, neutrophils, lymphocytes, C-reactive protein (CRP), and carcinoembryonic antigen (CEA).

Peripheral blood smears were examined microscopically for morphology, while serum CRP and CEA concentrations were determined using standard laboratory assays.

#### 2.2.3. Statistical Analysis

All statistical analyses were performed using the R statistical environment (version 4.0.0) [26]. We also used the “cluster” statistical package” (Version 2.1.0. CRAN) [27].

The use of linear progression and causal relationships was not appropriate in the context of the analysed clinical phenomena. Therefore, the study was extended to include taxonomic analysis [28]. The formation of patient subgroups based on the principles of taxonomy was determined by the similarity of specific clinical and laboratory parameters.

The Marczewski–Steinhaus metric [29] was used to measure pairwise taxonomic distances between patients, defined as: d(A, B) = ∣AΔB∣ max (∣A∣, ∣B∣).

The use of this metric allowed a structural interpretation of relationships between patients considered as sets of features. The selection of this metric was motivated by the mixed nature of the dataset, which included both continuous and pseudo-categorical variables, making it suitable for assessing structural similarities between patients, especially for capturing heterogeneous biomedical traits.

Complete-linkage agglomeration was applied because it generates compact, internally consistent clusters, which is important for the clinical interpretation of patient phenotypes.

Hierarchical clustering was performed using the complete-linkage agglomeration method, defined as:

$D(X,Y)=\underset{x\in X,y\in Y}{\max}d(x,y)$ [30]

A total of 84 trivariate combinations of variables were evaluated during the exploratory phase. Cut-off thresholds for dendrogram segmentation were determined using a scree-plot criterion. Two taxonomies demonstrated the highest internal coherence and clinical interpretability:

1. BMI + neutrophils + platelets,
2. age + lymphocytes + platelets.

Only these two taxonomic models were retained in the final results of the manuscript for clarity. As the taxonomic analysis was exploratory in nature, no correction for multiple comparisons (Bonferroni or FDR) was applied, and the findings should be regarded as hypothesis-generating.

#### 2.2.4. Limitations of the Study

This study has several limitations that should be acknowledged.

First, the analysis was cross-sectional and did not include survival or treatment-response data (e.g., overall or disease-free survival). Therefore, the prognostic value of the identified clusters should be interpreted cautiously until confirmed in prospective cohorts.

Second, although ordinal logistic regression was used to assess the association between clinical and hematological parameters and tumour stage, protein-level validation of the analysed markers was not performed due to the retrospective design of the study.

Third, the taxonomic analysis included multiple trivariate combinations (n = 84), which increases the probability of type I error. Given the exploratory nature of this approach, no correction for multiple comparisons (Bonferroni or FDR) was applied; hence, these findings should be considered hypothesis-generating rather than confirmatory.

Fourth, although sex-related differences were addressed in the statistical models, not all results were stratified by sex due to limited subgroup sizes.

Finally, this study was based on data from a single academic centre, which may limit generalizability to other populations.

## 3. Results

### 3.1. Study Group

The study group included 111 women (mean age 64.94 years; range 31–90 years). The mean height was 161.2 cm (range 143–176 cm), and the mean weight was 69.3 kg (range 32–140 kg). The mean BMI in women was 25.6 kg/m^2^. The study also included 118 men (mean age 63.98 years; range 36–94 years); the mean height was 174.1 cm (range 149–189 cm), and the mean weight was 81.6 kg (range 50–160 kg). The mean BMI was 26.9 in men—Table 1.

### 3.2. Laboratory and Anthropometrics Parameters in Study Group

The analysis of laboratory and anthropometric parameters in the study groups showed different results depending on the gender. When laboratory parameters of the female subgroup were analysed, the mean values were as follows: haemoglobin—11.99 g/dl, leukocytes—8.04, neutrophils—5.82, lymphocytes—1.64, platelets—311.2 and CEA—81.31. The mean values of PLR and NRL were 221.54 and 4.23, respectively. In turn, the results of the laboratory findings in the male subgroup were as follows: haemoglobin—12.41 g/dl, leukocytes—8.6, neutrophils—6.26, lymphocytes—1.63, platelets—316.7, CEA—132.78, PLR—234.52 and NRL—4.74.

The mean haemoglobin level in women was 11.99 g/dl and 12.41 g/dl in men, which might have been due to physiological differences between the genders. Low haemoglobin levels in women may be an indicator of a higher risk of anaemia, particularly in terms of cancer. The mean white blood cell counts in women and men were 8.04 and 8.6, respectively. The mean lymphocyte counts in women were 1.64 and 1.63 in men. The similar values of the parameters indicate the preservation of basic immune reactivity in both genders, although some differences in other blood parameters were found. The mean neutrophil counts were 5.82 in women and 6.26 in men. Higher neutrophil counts in men might have been associated with a more pronounced inflammatory response or might have reflected the differences in the immune response to cancer.

The mean platelet counts in women and men did not differ significantly, indicating a similarity in the blood coagulation response between both genders, which is essential in terms of the risk of developing thrombotic complications in CC. PLR showed no significant dimorphism, which is natural given the similar levels of platelets. The mean CEA in women was 81.31. However, it was significantly higher in men (132.78). High levels of CEA in men may indicate a higher risk of cancer progression and metastasis, as CEA is often associated with more aggressive forms of CC. Determination of NRL showed that the mean value in women was significantly lower than in men, which may have indicated a greater influence of inflammation on cancer progression since a high neutrophil-to-lymphocyte ratio is often associated with a poor prognosis in cancer patients.

### 3.3. Nosological Structure

**Figure 1:**
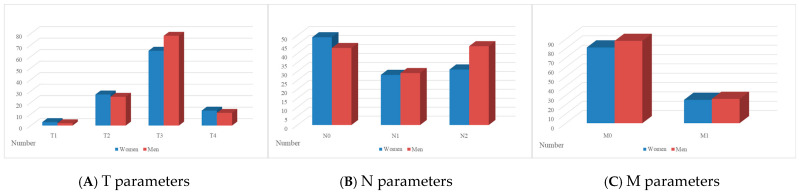
(**A**–**C**) Nosological structure of the disease according to TNM parameters (**A**—T, **B**—N, **C**—M) in female and male subgroups.

The nosological structure also demonstrated some sex-related dimorphism. Differentiation of patients according to the TNM classification showed that, among women, the following subgroups were identified: T1—3 patients, T2—27, T3—65, T4—13; N0—49, N1—28, N2—31; M0—81 and M1—25 patients. Among men, the corresponding distribution was: T1—2, T2—25, T3—78, T4—11; N0—43, N1—29, N2—44; M0—88 and M1—26 patients. The comparison of the nosological structure is presented in Figure 1.

The interpretation of the above data shows that although the overall trends in the distribution of disease stages were similar between the genders, some differences were found. For instance, a higher proportion of women presented with T3 tumours, while a higher proportion of men presented with T3 and N2 tumours. A slight increase in the number of men with distant metastases (M1) was also found compared to women. The data may indicate the need for a more differentiated approach to the diagnosis and treatment of CRC, depending on the gender, given their specific characteristics in the clinical course of the disease.

Statistically significant results (*p* < 0.05) and the results approaching significance (*p* < 0.1) obtained by ordinal logistic regression are given in Table 2. This allowed us to better understand the potential impact of different biomarkers on the course and prognosis of CRC.

**Figure 2:**
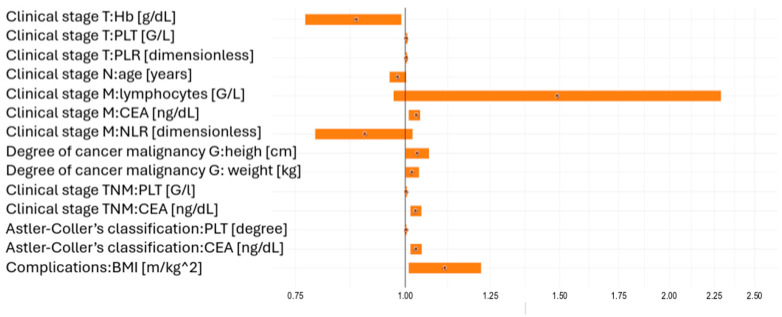
Odds ratios illustrating the impact of statistically significant (*p* < 0.05) and borderline significant (*p* < 0.1) risk factors on clinical tumour stage (TNM), tumour grade (G), Astler–Coller classification, and clinical complications (improvement vs. deterioration).

The statistical interpretation of the data in Table 2 showed that increased haemoglobin concentration was negatively correlated with the progression of the clinical stage (T stage). An increase in the blood level by 1 g/dL correlated with a decreased risk of progression according to the TNM classification by about 12%, while an increase by 5 g/dl correlated with a reduced progression risk by about 47%. Therefore, an inverse proportional relationship was found. In turn, platelet counts and PLR levels were directly correlated with disease progression (T parameter). The statistical interpretation of other results included in Table 2 was similar. The statistical results were presented in parallel on the forest plot shown in Figure 2.

The method used here was based on the framework of logistic regression with a dichotomous response variable and represents its extension to a scale with more than two categories [31]. In ordinal logistic regression, the dependent (outcome) variable has an ordered scale, and the estimated regression parameters are interpreted in the same way as in standard logistic regression, i.e., by means of odds ratios (ORs).

However, adopting a linear dependence model and the causal relationships obtained under this assumption was not fully satisfactory in substantive terms for the clinical phenomena under analysis. Therefore, the statistical analysis was extended by an alternative, methodologically distinct approach to the data in the form of a taxonomic analysis.

### 3.4. Characteristics of Taxonomy Types

Next, hierarchical clustering was performed using the Marczewski–Steinhaus metric and the complete-linkage agglomeration method. A total of 84 taxonomic sets of clinical features were analysed, of which two were considered most informative and clinically interpretable: Taxonomy I (BMI, neutrophil count, platelet count) and Taxonomy II (age, lymphocyte count, platelet count). These two combinations yielded the highest number of statistically significant associations (*p* < 0.05) and results at the threshold of statistical significance (*p* < 0.1) with clinical responses such as T, N, M, overall TNM stage, and Astler–Coller classification.

#### 3.4.1. Taxonomy I

Based on BMI, neutrophils, and PLT, three main patient types (“Type 1”, “Type 2” and “Type 3”) were identified. Group comparisons between these types were performed using one-way ANOVA or the Kruskal–Wallis test, depending on the distribution of variables. Statistically significant (*p* < 0.05) and borderline (*p* < 0.1) differences are presented in Table 3.

**Figure 3:**
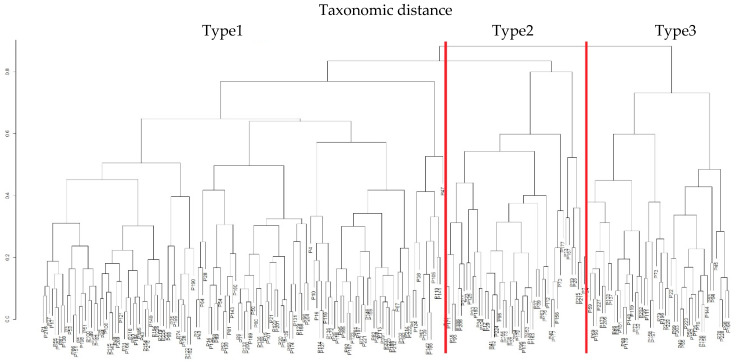
Taxonomic dendrogram and forest plot illustrating the three patient types identified in Taxonomy I based on BMI, neutrophil count, and platelet count (PLT).

The clinical and laboratory profiles corresponding to the three clusters in Taxonomy I are illustrated in Figure 3.

In summary, the statistical analysis shows that the three patient types identified in Taxonomy I are characterised by distinct relative levels of features and clinical responses (Table 4). In the most extreme cases, patients with low levels of clinical response in terms of T, N, M, overall TNM stage, and Astler–Coller classification were relatively characterised by:

- a moderate BMI,
- a high neutrophil count,
- a moderate PLT level,
- a moderate body weight,
- a moderate haemoglobin level,
- a high WBC count,
- a moderate CEA concentration,
- a high NLR, and
- a moderate PLR.

In contrast, patients with the poorest prognosis were those relatively characterised by:

- a low BMI,
- a moderate neutrophil count,
- a high PLT count,
- a low body weight,
- a low haemoglobin level,
- a moderate WBC count,
- a high CEA concentration,
- a moderate NLR, and
- a high PLR.

Based on statistical comparisons, Type 3 patients had the poorest prognosis and were characterised by, low BMI, mean neutrophil counts, high platelet counts, low weight, low haemoglobin levels, mean leukocyte counts, high CEA concentrations and mean NLR and PLR levels—Table 4.

#### 3.4.2. Taxonomy II

**Figure 4:**
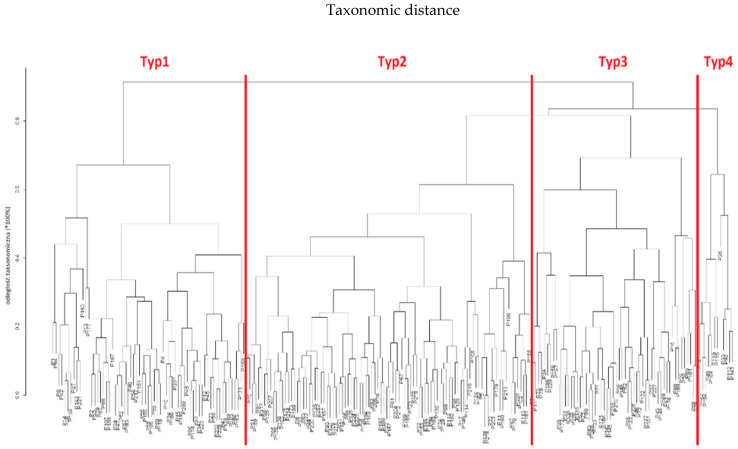
Taxonomic dendrogram of patients and their profiles based on age, lymphocyte count and platelet count (PLT) in Taxonomy II.

Using the same computational procedures, Taxonomy II was constructed based on patient age, lymphocyte count and platelet count (PLT). In this feature space, no equally clear “harmonic” ordering of data and inter-variable dependencies was observed as in Taxonomy I; however, the clustering still yielded four patient types (“Type 1”, “Type 2”, “Type 3” and “Type 4”). The clinical characteristics of these four types are presented in Table 5 and illustrated in Figure 4.

When a similar analysis was conducted in patients included in Taxonomy II, no similar homogeneity of data was found. Nevertheless, the group was divided into four types (“Type 1”, “Type 2”, “Type 3” and “Type 4”). The clinical characteristics of Taxonomy II, including the four identified types, are shown in Table 5.

The analysis of the data using the same analytical scheme as in the case of Taxonomy I showed that the most representative type in Taxonomy II was Type 2 (n = 96). These patients exhibited the highest levels of lymphocytes, haemoglobin, and leukocytes, along with the lowest levels of NLR and PLR. They also had a relatively lower clinical stage according to the TNM and Astler-Coller classification.

Importantly, Type 2 patients differed significantly in terms of clinical parameters from Type 1 subjects of the same age, although both groups represented a similar health status as measured by the oncological classification. The age of the patients was inversely proportional to their height. The statistical analysis showed that younger patients had a worse prognosis. The study also showed some other indirect causal relationships, which were relatively insignificant compared to the above findings.

The results of taxonomies for other combinations of risk factors that provided at least one statistical difference (*p* < 0.05) or borderline statistical significance (*p* < 0.1) in terms of clinical responses (TNM classification) are given in Table 6.

The analysis of the data for the additional taxonomic sets showed that only two of them reflected causal relationships that were considered reliable due to the number of positive clinical responses. Therefore, 84 combinations of clinical features were analysed. Only 20 (2 + 18) taxonomic combinations reflected a causal relationship with the clinical responses, such as stages (T, N, M). Based on the taxonomic analysis, the most important prognostic factors for patients in terms of oncological classification were BMI, lymphocyte, platelet and haemoglobin levels. The taxonomic relationships identified here supplement the knowledge about causal patterns obtained from ordinal logistic regression, in which these factors were expressed through odds ratios, providing a structural and nonlinear perspective on the prognostic value of these factors.

## 4. Discussion

This study analysed systemic inflammatory, haematological, and anthropometric parameters in patients with newly diagnosed colorectal cancer (CRC), with particular emphasis on sex-related differences and tumour stage. Male patients demonstrated consistently higher neutrophil and leukocyte counts, elevated CEA concentrations, and higher NLR values, which may reflect both a more pronounced inflammatory response and potentially more aggressive tumour biology. These observations are in line with previous studies suggesting sex-specific biological and clinical patterns in CRC progression, where obesity and elevated BMI show a stronger correlation with CRC incidence in men compared with women [32,33,34,35,36].

Multivariable logistic regression confirmed that PLR and CEA levels were positively correlated with more advanced TNM stages, while higher haemoglobin concentration was inversely associated with tumour progression. These findings reinforce the prognostic value of accessible and cost-effective systemic inflammatory markers. Such markers, particularly PLR and NLR, have been consistently linked to adverse outcomes in CRC [37,38] and other malignancies, including lung [39,40], gastric [41,42], and ovarian cancers [43,44], as well as in patients undergoing immunotherapy [45].

The novel contribution of this study lies in the introduction of a taxonomy-based classification system that integrates inflammatory, haematological, and anthropometric data. Using a multivariate clustering approach, three distinct clinical-biochemical taxa (Taxonomy I) were identified, each with a unique systemic and metabolic profile. Patients classified as Type 3 exhibited the most unfavourable characteristics: low BMI and haemoglobin levels, high platelet counts, elevated CEA and PLR values, and advanced tumour stage (TNM IIIC). This group likely represents a cachectic-inflammatory phenotype associated with poor prognosis. In contrast, Type 1 patients had higher BMI, lower inflammatory markers, and less advanced disease, while Type 2 was characterised by elevated neutrophil and leukocyte counts, indicating a systemic inflammatory response.

A secondary classification (Taxonomy II) further stratified the cohort into four groups. Type 2 patients in this model displayed the most favourable profiles, with high lymphocyte and haemoglobin levels, low NLR and PLR values, and less advanced clinical staging. These taxonomic patterns suggest that inflammatory and metabolic markers can be used not only as prognostic indicators but also as a basis for stratifying patients into biologically and clinically meaningful subgroups.

Our results are consistent with the growing body of evidence linking systemic inflammation and nutritional status to cancer prognosis. For instance, Petrelli et al. [34] reported that while obesity is generally associated with decreased overall and cancer-specific survival, it may paradoxically confer a survival advantage in certain malignancies such as lung, kidney, and melanoma. Similarly, Harmantepe et al. [46] proposed combining clinical, inflammatory, and histopathological indicators in obstructive CRC to refine survival prediction. Our taxonomy reflects this complexity by integrating body composition with immunological and haematological parameters.

The prognostic significance of PLR and NLR has been well documented across tumour types. Zhou et al. [47] demonstrated that high PLR was associated with worse outcomes in immunotherapy-treated lung cancer, while Wang et al. [48] reported a similar association with clinical outcomes in surgically treated lung cancer patients. Unlike previous studies focusing on isolated markers, our taxonomy combines these parameters into multidimensional and clinically interpretable subtypes. Importantly, systemic inflammatory markers such as NLR and PLR have also been linked to surgical outcomes, including anastomotic leakage in both general and elderly CRC populations [49,50,51,52,53,54].

The generalisability of this taxonomic framework to other malignancies appears promising and warrants further investigation. Although the present analysis focuses on CRC, stratification of patients based on routinely collected inflammatory, haematological, and anthropometric data could also enhance prognostic precision in other oncological settings.

Nevertheless, several limitations should be acknowledged. The double-centre, cross-sectional design without survival data precludes direct conclusions regarding long-term outcomes. Further multicentre prospective studies are required to validate these taxonomies, integrate molecular tumour characteristics, and assess their predictive value for treatment response and survival.

## 5. Conclusions

Colorectal cancer patients can be systematically classified into clinical-biochemical taxonomies that reflect distinct inflammatory and nutritional states, which correspond to differences in tumour advancement. Given their accessibility and low cost, the proposed taxonomy-based approach has potential for implementation in routine clinical practice and may support risk-adapted management strategies in CRC and, potentially, other malignancies. Such a taxonomy may facilitate early identification of high-risk patients, particularly those with a cachectic-inflammatory phenotype, who could benefit from intensified surveillance or adjunctive nutritional and anti-inflammatory interventions. Future prospective multicentre studies are needed to validate these findings, determine their predictive value for survival and treatment response, and explore integration with molecular and genomic tumour characteristics to further refine patient stratification.

## Figures and Tables

**Table 1:** Characteristics of Patients.

Parameter	Women (n = 111)	Men (n = 118)
Age (years)	Mean: 64.94	Mean: 63.98
	Range: 31–90	Range: 36–94
Height (cm)	Mean: 161.2	Mean: 174.1
	Range: 143–176	Range: 149–189
Weight (kg)	Mean: 69.3	Mean: 81.6
	Range: 32–140	Range: 50–160
BMI (kg/m^2^)	Mean: 25.6	Mean: 26.9

**Table 2:** Statistically significant results were obtained using ordinal logistic regression (*p* < 0.05 and *p* < 0.1).

Clinical Parameter	Risk Factor	OR	95% Probability	*p*
Stage T	Haemoglobin [g/dL]	0.88	(0.77;0.99)	0.045
Stage T	platelets [g/g]	1.003	(1.001;1.006)	0.004
Stage T	PLR	1.003	(1.001;1.005)	0.003
Stage N	Age [years]	0.981	(0.960;1.003)	0.088
Stage M	lymphocytes [g/L]	1.49	(0.97;2.29)	0.070
Stage M	CEA [ng/dL]	1.03	(1.01;1.04)	<0.001
Stage M	NRL	0.90	(0.79;1.02)	0.099
Malignancy grade (G)	height [cm]	1033	(1.002;1.064)	0.035
Malignancy grade (G)	weight [kg]	1.019	(1001;1037)	0.034
Clinical TNM stage	platelets [g/g]	1.003	(1.001;1.005)	0.005
Clinical TNM stage	CEA [ng/dL]	1028	(1.014;1.043)	0.000
Astler-Coller classification (stage)	platelets [g/g]	1.002	(1000;1004)	0.018
Astler-Coller classification (stage)	CEA [ng/dL]	1029	(1.014;1.044)	<0.001
Complications	BMI [m/kg^2^]	1.11	(1.01;1.22)	0.026

**Table 3:** Clinical and laboratory characteristics of the three taxonomic patient types identified in Taxonomy I.

Risk Factor	Type 1	Type 2	Type 3	*p*
BMI (m/kg^2^)	(131), 28.6 ± 5.1; 27.8	(43), 25.4 ± 5.3; 25.3	(47), 23.1 ± 4.9; 22.7	<0.0001
Neutrophils (g/L)	(132), 4.71 ± 1.60; 4.7	(45), 9.44 ± 3.06; 9.3	(48), 6.52 ± 2.09; 6.3	<0.0001
Platelets (g/L)	(134), 267 ± 69; 264	(46), 292 ± 103; 300	(49), 462 ± 137; 448	<0.0001
Weight (kg)	(131), 80 ± 16; 77	(43), 71 ± 16; 73	(47), 66 ± 16; 63	<0.0001
Haematocrit (g/dL)	(134), 12.8 ± 1.9; 13.1	(46), 11.7 ± 2.4; 11.5	(49), 11.1 ± 1.8; 11	<0.0001
Leukocytes (g/L)	(133), 7.22 ± 2.24; 6.9	(46), 10.8 ± 3.52; 11.4	(49), 9.05 ± 2.49; 9	<0.0001
CEA (ng/dL)	(85), 15.1 ± 36.2; 3.1	(34), 143 ± 770; 3.5	(25), 368 ± 1517; 7	0.0998
NRL	(132), 3.31 ± 1.85; 2.8	(45), 8.13 ± 5.16; 6.9	(48), 4.35 ± 2.23; 3.8	<0.0001
PLR	(132), 192 ± 105; 169	(45), 242 ± 141; 199	(48), 315 ± 170; 253	<0.0001
T (stage)	(132), 2.8 ± 0.6; 3	(45), 2.6 ± 0.8; 3	(48), 3.0 ± 0.5; 3	0.0037
N (stage)	(131), 0.9 ± 0.9; 1	(45), 0.7 ± 0.8; 1	(48), 1.1 ± 0.9; 1	0.0990
M (stage)	(129), 0.2 ± 0.4; 0	(45), 0.1 ± 0.3; 0	(45), 0.3 ± 0.5; 0	0.0193
Clinical TNM stage	(128), 4.1 ± 2.3; IIIB	(44), 3.6 ± 2.1; IIIA	(45), 5.0 ± 2.0; IIIC	0.0064
Astler-Coller classification (stage)	(128), 3.3 ± 1.5; C2	(45), 2.9 ± 1.4; C1	(45), 3.8 ± 1.2; C2	0.0076

**Table 4:** Summary of clinical parameter levels by Taxonomy I type.

Risk Factor/Clinical Response	Type 1	Type 2	Type 3
BMI	High	Mean	Low
Neutrophils	Low	Low	Mean
Platelets	Low	Mean	High
Weight	High	Mean	Low
Haemoglobin	High	Mean	Low
Leukocytes	Low	High	Mean
CEA	Low	Mean	High
NRL	Low	High	Mean
PLR	Low	Mean	High
T stage	Mean	Low	High
N stage	Mean	Low	High
M stage	Mean	Low	High
Clinical TNM stage	Mean	Low	High
Astler-Coller classification (stage)	Mean	Low	High

**Table 5:** Clinical characteristics of taxonomy II with the types 1–4.

Risk Factor/Clinical Parameter	Type 1	Type 2	Type 3	Type 4	*p*
Age	(65), 70 ± 8; 70	(96), 68 ± 8; 68	(56), 57 ± 8; 57	(12), 40 ± 5; 41	<0.0001
Lymphocytes (g/L)	(65), 0.97 ± 0.27; 1.00	(94), 2.21 ± 0.63; 2.15	(54), 1.42 ± 0.44; 1.42	(12)—1.69 ± 0.42; 1.60	<0.0001
Platelets (g/L)	(65), 262 ± 102; 234	(96), 290 ± 81; 285	(56), 422 ± 144; 373	(12), 280 ± 81; 283	<0.0001
Height (cm)	(62), 165 ± 11; 166	(93), 167 ± 9; 167	(54), 170 ± 10; 169	(12), 173 ± 8; 173	0.0122
Haemoglobin (g/dL)	(65), 11.6 ± 2.3; 12.0	(96), 12.9 ± 1.9; 13.1	(56), 11.7 ± 1.9; 11.6	(12), 12.2 ± 2.3; 12.7	0.0004
Leukocytes (g/L)	(65), 7.44 ± 3.44; 6.67	(95), 8.97 ± 2.45; 8.90	(56), 8.38 ± 3.04; 8.25	(12), 8.09 ± 2.55; 7.55	0.0146
NRL	(65), 6.43 ± 4.73; 5.25	(94), 2.97 ± 1.52; 2.64	(54), 4.92 ± 2.85; 4.52	(12), 4.09 ± 2.78; 3.11	<0.0001
PLR	(65), 296 ± 155; 248	(94), 140 ± 45; 135	(54), 315 ± 136; 268	(12), 169 ± 45; 174	<0.0001
T stage	(64), 2.8 ± 0.6; 3	(96), 2.8 ± 0.7; 3	(53), 3.1 ± 0.5; 3	(12)—2.5 ± 0.7; 3	0.0059
N stage	(64), 0.8 ± 0.8; 1	(95), 0.8 ± 0.9; 1	(53), 1.2 ± 0.8; 1	(12)—1.4 ± 0.8; 2	0.0097
M stage	(64), 0.1 ± 0.3; 0	(93), 0.3 ± 0.5; 0	(50)—0.2 ± 0.4; 0	(12)—0.3 ± 0.5; 0	0.0877
Clinical TNM stage	(64), 3.8 ± 2.1; IIIB	(91), 4.0 ± 2.4; IIIA	(50)—5.0 ± 1.7; IIIB	(12)—5.0 ± 2.0; IIIB	0.0218
Astler-Coller classification (stage)	(64), 3.0 ± 1.4; C2	(92), 3.1 ± 1.6; C2	(50), 3.8 ± 1.0; C2	(12)—3.7 ± 1.3; C2	0.0194

**Table 6:** Three variables of a taxonomic set of clinical features that predicted statistically different (*p* < 0.05) clinical responses.

Statistical Significance (*p* < 0.05) for	Parameter 1	Parameter 2	Parameter 3
2 or 3 clinical responses	BMI	Lymphocytes	Platelets
	BMI	Lymphocytes	PLR
1 clinical response	Age	BMI	Lymphocytes
	Age	BMI	NRL
	Age	Leukocytes	Lymphocytes
	Age	Lymphocytes	NRL
	BMI	Haemoglobin	Neutrophils
	BMI	Haemoglobin	Platelets
	BMI	Leukocytes	NRL
	BMI	Neutrophils	NRL
	Haemoglobin	Leukocytes	Neutrophils
	Haemoglobin	Leukocytes	Lymphocytes
	Haemoglobin	Leukocytes	Platelets
	Haemoglobin	Neutrophils	Platelets
	Haemoglobin	Lymphocytes	Platelets
	Haemoglobin	Lymphocytes	NRL
	Neutrophils	Lymphocytes	NRL
	Neutrophils	Lymphocytes	PLR

## Data Availability

Data may be made available from the corresponding author upon reasonable request.
